# Impact of ophthalmic clinical service use in mitigating myopia onset and progression in preschool children: a retrospective cohort study

**DOI:** 10.1186/s12886-024-03488-5

**Published:** 2024-05-27

**Authors:** Pingping Lyu, Jingwen Hu, Yujie Wang, Jingjing Wang, Xiangui He, Huijing Shi

**Affiliations:** 1 Department of Maternal, Child and Adolescent Health, School of Public Health, Fudan University, 138 Yixueyuan Road, Xuhui District, Shanghai, 200032 China; 2 Shanghai Eye Disease Prevention and Treatment Center, Shanghai Eye Hospital, Shanghai, China

**Keywords:** School vision screening, Myopia onset, Progression, Health service, Referral, Cox proportional model

## Abstract

**Background:**

Although school screenings identify children with vision problems and issue referrals for medical treatment at an ophthalmic hospital, the effectiveness of this approach remains unverified.

**Objective:**

To investigate the impact of ophthalmic clinical services on the onset and progression of myopia in preschool children identified with vision impairment.

**Methods:**

Using data from the Shanghai Child and Adolescent Large-scale Eye Study (SCALE), this retrospective cohort study evaluated the visual development of children from three districts—Jing’an, Minhang, and Pudong—which are representative of geographic diversity and economic disparity in Shanghai’s 17 districts. Initially, in 2015, the study encompassed 14,572 children aged 4–6 years, of whom 5,917 needed a referral. Our cohort consisted of 5,511 children who had two or more vision screenings and complete personal information over the follow-up period from January 2015 to December 2020. We divided these children into two groups based on their initial spherical equivalent (SE): a High-risk group (SE > -0.5 D) and a Myopia group (SE ≤ -0.5 D). Within each of these groups, we further categorized children into Never, Tardily, and Timely groups based on their referral compliance to compare the differences in the occurrence and progression of myopia. Cox proportional models were applied to estimate hazard ratios (HRs) for myopia incidence per person-years of follow-up in High-risk group. Generalized additive models(GAM) was used to calculating the progression for annual spherical equivalent changes in all children.

**Results:**

Of the 5,511 preschool children (mean age, 5.25 years; 52.24% male) who received a referral recommendation, 1,327 (24.08%) sought clinical services at an ophthalmic hospital. After six years of follow-up, 65.53% of children developed myopia. The six-year cumulative incidence of myopia in the Never, Tardily, and Timely groups was 64.76%, 69.31%, and 57.14%, respectively. These percentages corresponded to hazard ratios (HRs) of 1.31 (95% CI, 1.10–1.55) for the Tardily group and 0.55 (95% CI, 0.33–0.93) for the Timely group, compared with the Never group. The HRs were adjusted for age, sex, and SE at study entry. Interestingly, the Timely group showed significantly less SE progression than the other groups (*P* < 0.001), and SE progression was higher in the High-risk group (-0.33 ± 0.37D/year) than in children with myopia (-0.08 ± 0.55D/year).

**Conclusion:**

Timely utilization of ophthalmic clinical services among children aged 4 to 6 years who fail school vision screenings can significantly reduce the incidence of myopia and slow SE progression.

## Introduction

Myopia, broadly accepted as one of the most prevalent ocular diseases, presents a concern of international magnitude [1–3]. A predictive study suggests that by the year 2050, myopia will afflict approximately 4758 million individuals, constituting nearly 49.8% of the global population [4]. There is a high prevalence of myopia, 80–90%, in young adults in East Asia [5]. Particularly in China, forecasts indicate that by the year 2050, the prevalence of myopia is expected to reach an estimated 84% among children and adolescents ages 3 to 19 years [6]. The foremost strategies to prevent the onset and slow the progression of myopia primarily involve public health initiatives, pharmacological approaches, and optical devices [7], such as increased outdoor time [8–11], reducing activities done at a short working distance [12–14], Chinese eye exercises [15, 16], low-dose atropine [17, 18], and repeated low-level red-light (RLRL) [19]. In China, the primary focus of prevention strategies lies in curbing the incidence of myopia, with secondary and tertiary prevention measures aimed at decelerating the progression of the condition enforced as a governmental imperative. Given the escalating prevalence of high myopia and subsequent severe complications, the deployment of primary prevention tactics are of utmost importance and are privileged within the existing national myopia control plan in China [20]. The distinct features of the myopia epidemic underscore the principal challenges: ensuring timely correction for myopic children during their developmental phase. This necessitates the implementation of efficacious school screening programs and expedited referrals for optical correction and myopia management [21].

The Shanghai Child and Adolescent Large-scale Eye Study (SCALE) is a citywide, prospective survey conducted throughout schools, aimed at addressing ocular health concerns in children aged between 4 and 14 years across Shanghai. In instances where school vision screenings detected abnormal visual acuity or refraction, a referral was initiated. This enabled children, under parental guidance, to visit hospitals for more intensive re-examinations of visual acuity, cycloplegia, and subjective refraction. These assessments were performed by experienced ophthalmologists or optometrists skilled in the diagnosis and treatment of ocular conditions within Shanghai, China. All pertinent study data were diligently compiled within an electronic database [22]. The utilization of health services has been proved to be effective in controlling of many diseases, such as smoking [23], early cancer [24], and chronic disease [25]. Nonetheless, only a handful of studies have influence of referral on the occurrence and progression of myopia. Myopia progression is irreversible and there is no cure [26]. The significance of curbing and managing myopia during early childhood cannot be understated. It is also well recognized that myopia occurring at an earlier age in childhood is associated with a faster myopia progression and higher degree of myopia in adult life [5, 27–29]. Hence, our study aims to investigate the impact of utilizing ophthalmic clinical health services post-school vision screening, on mitigating the onset and progression of myopia among preschool children.

## Methods

### Data source and study population

Our research was based on the SCALE study. SCALE represents the most extensive investigation into the prevalence of myopia among the Shanghai children and adolescent population aged 4–14. Since 2012, community doctors have annually examined children and adolescents from all 17 districts and counties in Shanghai, China. The Detailed methods of the study were previously reported [22]. In brief, if a child’s visual acuity or refraction was determined to be abnormal during the school vision screening, the child would fail the screening and would be recommended for further evaluation. Students who failed the screening would receive a paper referral notice, but they were not compelled to do so, nor did they receive further phone calls or text message reminders.

According to the referral criteria in the SCALE study, children meeting the following conditions were encouraged to visit the designated hospital for further examination and, if necessary, management:1) The uncorrected visual acuity of either eye is abnormal(for ages 4–5 years, UCVA < 0.6, and for age 6 years, UCVA < 0.8). 2)The uncorrected visual acuity is normal but have a high risk of myopia according to the spherical equivalent (SE). These children have normal UCVA of both eyes (for 4–5 years, UCVA ≥ 0.6, for 6 years, UCVA ≥ 0.8) and SE of either eye < 0.00D. These children will also need regular referrals.

**Fig. 1:**
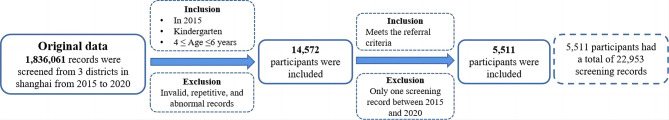
Flowchart for selecting the study subjects

Since 2015, the data quality of the Shanghai Child and Adolescent Large-scale Eye Study (SCALE) has significantly improved. Owing to the extensive size of the database, we selected Jing’an, Minhang, and Pudong districts for our study. These districts represent the geographic location and economic disparities amongst Shanghai’s 17 districts. Figure 1 represents the process of selecting the study subjects.

### Definition

Spherical equivalent (SE) was defined as the sum of the spherical power and half of the cylinder. Myopia was determined as a SE of -0.5D or less without cycloplegia. Based on the initial SE, children were categorised into two groups for a six-year observational study on visual development: the High-risk group (SE > -0.5D) and the Myopia group (SE ≤ -0.5D). As per the guidelines in Shanghai, students failing school vision screenings are recommended to seek ophthalmic clinical referral services within three months. Accordingly, we further bifurcated each subgroup into three groups, based on the compliance with this referral recommendation: Never group—children who did not seek ophthalmic clinical referral services during the six-year follow-up period; Tardily group—children who sought such services one or more times between three months post the initial vision screening and the end of the follow-up period; and Timely group—children who promptly sought ophthalmic clinical referral services within three months post referral recommendation issuance.

### Data analysis

The statistical analyses for this study were performed using SAS version 9.4, and Sigmaplot 14.0 was employed for generating graphical figures. Normality of the data sets was assessed using the Kolmogorov-Smirnov test. Despite some variables conforming to the normal distribution, due to the large sample size, we expressed results as mean ± standard deviation (SD) or as a percentage. Depending on the data type and distribution, the age and sex among the groups were compared employing the Kruskal-Wallis test, Fisher’s exact test, and the χ^2^ test. The screening frequency and spherical equivalent (SE) were compared using the Kruskal-Wallis test and analysis of variance. In this cohort study, the incidence of myopia was calculated per person-years of follow-up. Cox proportional hazard models were utilized to estimate hazard ratios (HRs) and 95% confidence intervals (CIs) for the incidence of myopia per referral compliance group, with non-referred children serving as the reference. Initially, unadjusted models were calculated, followed by fully adjusted models accounting for age, sex, and initial SE. The dependent variable of this study is non normal data, the mean rate of change in SE was compared among children with different initial age, sex and SE by using generalized additive models(GAM). The utilization of Generalized Additive Models (GAM) facilitated error pattern specification and proved an adequate fit for datasets with non-normal distributions, yielding lower and more reliable *p*-values [30]. Since our data is non-normal and the relationship between dependent and independent variables is also not linear, GAM provides a more effective analytical method than traditional linear models.

## Results

### Baseline characteristics

Table 1 presents the baseline characteristics of the 5,511 preschool children included in this study, categorized according to referral compliance. While the compliance rate was 24.08%, the proportion of timely referrals stood at a mere 2.03% (112 out of 5,511). There was no significant difference in compliance rates between the High-risk and Myopia groups (χ^2^ = 0.14, *P* = 0.71). The study children had a mean age of 5.25 ± 0.75 years, with 52.24% being boys. The mean spherical equivalent (SE) was − 0.87 ± 1.29D. At the outset of the follow-up period, there were differences in age and SE among children in each subgroup. However, the sex distribution within the Myopia group showed no significant difference.

**Table 1:** Characteristics of the study cohort at baseline, according to referral compliance categories

		Total (*n* = 5511)	Never (*n* = 4184)	Tardily (*n* = 1215)	Timely (*n* = 112)	*P* Value
Total	Participants, n(%)	5511	4184(75.92)	1215(22.05)	112(2.03)	NA
	Compliant participants, n(%)	1327(24.08)	NA	NA	NA	NA
	Sex(%male)	2879(52.24)	2229(53.27)	596(49.05)	54(48.21)	0.024
	Mean age ± SD, y	5.25 ± 0.75	5.28 ± 0.75	5.20 ± 0.73	4.64 ± 0.64	< 0.001
	SE, mean ± SD	-0.87 ± 1.29	-0.88 ± 1.31	-0.86 ± 1.23	-0.65 ± 1.50	< 0.001
High-risk						
	Participants, n(%)	2437	1856(76.16)	518(21.26)	63(2.59)	NA
	Compliant participants, n(%)	581(23.84)	NA	NA	NA	NA
	Sex(%male)	1296(53.18)	998(53.77)	268(51.74)	30(47.62)	0.478
	Mean age ± SD, y	5.20 ± 0.75	5.17 ± 0.75	5.15 ± 0.72	4.63 ± 0.60	< 0.0001
	SE, mean ± SD	0.03 ± 0.85	0.03 ± 0.86	0.03 ± 0.83	0.23 ± 0.59	< 0.0001
Myopia						
	Participants, n(%)	3074	2328(75.73)	697(22.67)	49(1.59)	NA
	Compliant participants, n(%)	746(24.26)	NA	NA	NA	NA
	Sex(%male)	1583(51.50)	1231(52.88)	328(47.06)	24(48.98)	0.025
	Mean age ± SD, y	5.28 ± 0.76	5.31 ± 0.76	5.22 ± 0.74	4.65 ± 0.69	< 0.001
	SE, mean ± SD	-1.58 ± 1.13	-1.60 ± 1.14	-1.52 ± 1.05	-1.79 ± 1.55	0.006

### Risk estimates of the association between referral compliance and incident myopia

**Fig. 2:**
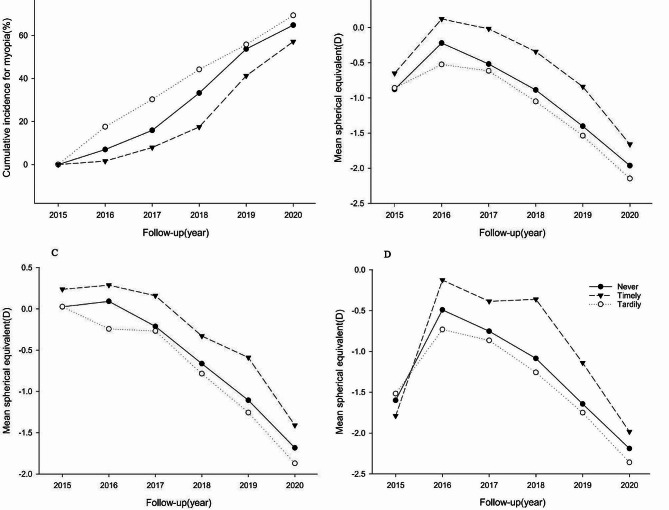
Line graphs showing the cumulative incidence for myopia among children with high risk (**A**); Mean SE change in all children(**B**), children with high risk (**C**) and children with myopia (**D**) during the 6 follow-up years by referral compliance

Among children at high risk for myopia, the mean follow-up period was 5.00 ± 0.94 years, with each child undergoing an average of 4.17 ± 1.25 examinations. Over the course of six years, 65.53% of children developed myopia. The six-year cumulative incidence of myopia was lowest in the Timely group (57.14%), compared to the Never (64.76%) and Tardily (69.31%) groups (Fig. 2, Picture A). The median survival times for the Never, Tardily, and Timely groups were 4.92 years (95% CI, 4.83–4.92), 4.50 years (95% CI, 4.00-4.92), and 5.83 years (95% CI, 4.42–5.92), respectively. A total of 1,597 incidences of myopia were recorded over 12,185 person-years. The incidence of myopia (per 1,000 person-years) differed according to referral compliance: 129.53 for the Never group, 138.34 for the Tardily group, and 118.42 for the Timely group (Table 2). When compared with children who never sought a referral, the hazard ratios (HRs) for cumulative incidence of myopia were 1.31 (95% CI, 1.10–1.55; *P* < 0.001) in the Tardily group and 0.67 (95% CI, 0.33–0.93; *P* < 0.05) in the Timely group.

**Table 2:** Shows the incidence and progression of myopia by subgroup

		Total	Never group	Delay group	Timely group
Risk					
	Participants, n(%)	2437	1856(76.16)	518(21.26)	63(2.59)
	Screening frequency, mean ± SD	4.2 ± 1.21*	4.10 ± 1.18	4.62 ± 1.24	4.16 ± 1.03
	Mean follow-up ± SD, y	5.00 ± 0.94*	5.00 ± 0.94	5.01 ± 0.92	4.83 ± 0.93
	Person-y of follow-up	12,185	9280	2595	304
	Incident myopia, n(%)	1597(65.53)	1202(64.76)	359(69.31)	36(57.14)
	Incidence myopia(per 10^–3^ person-y), 95% CI	131.06(125.07-137.06)	129.53(122.69-136.36)	138.34(125.05-151.64)	118.42(81.89-154.95)
	Median survival time, 95% CI	4.92(4.67–4.92)	4.92(4.83–4.92)	4.50(4.00-4.92)	5.83(4.42–5.92)
	Unadjusted HR, 95% CI	NA	1(Reference)	1.14(1.01–1.28)*	0.67(0.50–0.97)*
	Adjusted HR^a^, 95% CI	NA	1(Reference)	1.31(1.10–1.55)*	0.55(0.33–0.93)*
	SE change^b^(D/year)	-0.33 ± 0.37*	-0.32 ± 0.37	-0.37 ± 0.37	-0.28 ± 0.37
Myopia					
	Participants, n(%)	3074	2328(75.73)	697(22.67)	49(1.59)
	Screening frequency, mean ± SD	4.12 ± 1.27*	4.00 ± 1.24	4.62 ± 1.28	4.00 ± 1.06
	Mean follow-up ± SD, y	4.92 ± 0.95*	4.91 ± 0.96	4.95 ± 0.92	4.79 ± 0.90
	Person-y of follow-up	15,124	11,430	3450	235
	SE change^b^(D/year)	-0.08 ± 0.55*	-0.06 ± 0.57	-0.14 ± 0.50	0.07 ± 0.60

^a, b^ Adjusted for age, sex and SE at study entry. *Significant value: *P* < 0.05

### Risk estimates of the association between referral compliance and progression of myopia

Among all 2,437 participants at high risk of myopia, the average rate of change in the spherical equivalent (SE) was − 0.33 ± 0.37D/year. The annual SE progression in the Timely group (-0.28 ± 0.37D/year) was significantly lower than that of the Never group (-0.32 ± 0.37D/year) and the Tardily group (-0.36 ± 0.37D/year) at later ages (*P* = 0.006) (Fig. 2, Picture B). There was a rapid increase in SE among children at high risk of myopia, indicating a severe trend (Fig. 2, Picture C). Conversely, children in the Myopia group experienced an initial rebound followed by a decline over the six-year follow-up period (Fig. 2, Picture D). The mean SE progression in children who promptly utilized ophthalmic clinical services after school vision screening (0.07 ± 0.60D/year) was significantly slower compared to those who never sought referral (-0.06 ± 0.57D/year) or were tardy in doing so (-0.14 ± 0.50D/year) at later ages (*P* = 0.005) (Table 2).

## Discussion

In this extensive, prospective, school-based cohort study, we discovered that a timely utilization of ophthalmic clinical services post school vision screenings in preschool children aged 4 to 6 years with vision disorders can mitigate the onset and progression of myopia.

During the 2015–2020 period, 24.08% of the 5,511 preschool children who received referral notice following failed school vision screenings attended follow-up appointments at the hospital for additional examinations at least once within the six-year follow-up period. However, only a meagre 2.03% of children required to seek ophthalmic clinical services did so within the stipulated three months. To our knowledge, no other cohort studies have examined the correlation between ophthalmic clinical service utilization in preschool children and the subsequent development and progression of myopia. In our follow-up study, we found that student age, visual health status, family annual income, and the presence of referral prompts in schools or community health centers all impact the utilization of ophthalmic clinical health services. The primary factor contributing to the current low compliance is the absence of a robust referral system [31].

We found that children who were referred for eye examinations at preschool, but did so tardily, presented the highest risk for developing myopia—even outstripping those who never sought a referral. Generally, these children sought medical treatment after a long interval—on average, 555 days—following their referral recommendations. We hypothesize that although these children had previously utilized ophthalmic clinical services, their vision issues by that time were severe, resulting in higher myopia incidence and progression rates compared to other children.

Constrained by the challenge of insufficient cooperation and a low follow-up rate among young children, only a handful of studies have undertaken a longitudinal observation to evaluate the likelihood of myopia in preschool children [32]. Our study’s results provide groundwork for comparing myopia prevalence rates in preschool children. Myopia is commonly seen among school-aged children, whereas its prevalence is relatively low in children aged 6 years or younger. However, congruent with other longitudinal studies, we identified a higher rate of vision disorders among preschool children. In this study, we discovered that 35.08% of children aged between 4 and 6 years exhibited visual abnormalities at the commencement of the follow-up period. Further, 13.98% were in high-risk groups and 21.1% already had myopia. The prevalence rate of an Vision In Preschoolers (VIP) study-targeted vision disorders among all children aged 3–5 years with sampling weights incorporated was 21.4% [33]. There were 17.0% children aged 3 to 6 years identified in having the visual abnormalities in Hong Kong preschool and 6.32% were myopes [34]. The prevalence of myopia under 6 years in Germany was lower than 5.0%, and remained virtually unchanged over a period of approximately 10 years [35]. Among children with high risk of myopia, 65.53% develop myopia during 6 years of follow-up. A 6-year follow-up of the Sydney Myopia Study revealed a mean annual incidence of myopia of 2.2% in the younger cohort among 1765 children with a mean age of 6.7 years at baseline [36]. In previous reports, the myopia rate in China was generally higher than that of other countries [6, 37], and the subjects of this study were children who were already visually impaired. It has been reported that early achievement of emmetropia is a risk factor for subsequent progression to myopia [38]. Most importantly, the refractive data in this study are all obtained without cycloplegia. One current study limitation is the likelihood of overestimating myopia prevalence due to children’s refractive status being assessed without cycloplegia. Administering cycloplegia in children requires substantial effort and resources, which were unavailable in our protocol. Furthermore, significant parental resistance to cycloplegia exists in this region, making it unrealistic to execute the study within the preset timeframe and resource allocation required for cycloplegia. The focus of this study isn’t to pinpoint an accurate diagnosis of myopia, but to review the significance of using commonplace health services to control and prevent myopia during large population mass screenings. Despite the potential for overestimating myopia prevalence, the application of noncycloplegic refraction for school screenings is fitting, given its inherent capability to effectively identify all instances of myopia [39].

In this study, we documented the longitudinal shifts in spherical equivalent (SE) refractive errors among Chinese preschoolers diagnosed with visual impairments. The mean rate of SE change in high-risk children and those with myopia were − 0.33 ± 0.37D/year and − 0.08 ± 0.55D/year, respectively. The Northern Ireland Childhood Errors of Refraction (NICER) reported that the estimated annual median change of participants with 6–7 years old was − 0.23D over the six-year period [40]. Hu Y et al. [41] reported that the mean rate of SE change in the children aged 5.12 years(IQR, 4.12–5.76 years) with myopia was − 0.59-0.47D/year. Some studies have revealed that younger children or children with greater initial myopic refractive errors are at a greater risk of myopia progression [41–44], especially in school-aged children [45, 46]. In our study, the myopia progression rate was lower among children referred timely, and children with myopia at study entry. The research results of Hu Y et al. [41] and Shih YF, et al. [47] also showed that the risk of myopia progression does not increase with the severity of initial SE in preschool children. The refractive development of human eyes represents a dynamic process, as ocular biometric parameters undergo changes from birth, resulting in corresponding alterations in the refractive state [48]. These results may not be exactly comparable to our study findings because the age and refraction distributions of the study populations were different. Nonetheless, the findings suggest that preschool myopia likely follows a distinct evolutionary course. Undeniably, further study is required to fully understand the mechanism of myopic regression [47].

The mitigated occurrence and progression of myopia in the Timely referred group might be attributed to their timely utilization of ophthalmic health services. Such services encompass not only health education initiatives like enhancing outdoor activities and maintaining proper reading and writing posture but also corrective interventions including eyeglasses prescription and medication usage. Moreover, physicians often require perpetual vigilance and frequent follow-ups from parents. This study, along with several other population-based researches, conveys that children with elevated risk should receive preventative guidance and close monitoring for the onset of myopia, so that necessary therapeutic interventions can be timely introduced [2, 7].

Compared to the school vision screening program, the referral mechanism has not been given sufficient attention in Shanghai. Our results provide strong evidence for regions to continue promoting and implementing referral work, demonstrating the necessity of promoting the utilization rate of ophthalmic clinical health services. Additionally, population screening, clinical early diagnosis, and early treatment are all extremely important aspects of disease prevention strategies in the secondary prevention stage. This study confirms that both school vision screening and early utilization of clinical health services are indispensable for children’s visual development, and the utilization of ophthalmic clinical health services after school vision screening is time-sensitive. Therefore, this study suggests that Shanghai should further enhance its well-established community-based school vision screening and referral systems to create a comprehensive and advanced primary pediatric eye care system, similar to those found in countries with more refined models [49–51].

There have been several cross-sectional studies of prevalence of myopia in school-aged children [52–56], but few longitudinal studies on the incidence and progression of myopia in China. In this large-scale, prospective, school-based cohort study, we attempted to evaluate the effectiveness of ophthalmic clinical health services on myopia prevention and control in preschool children, which is highly innovative. We also acknowledge several weaknesses in this study. This study focuses on preschool children afflicted with visual disorders, perhaps not depicting a true representation of the preschool population with healthy vision. Irregularities were present in terms of the frequency and intervals of monitoring among children, which could potentially lead to the inaccurate recording of myopia onset during the follow-up period. Moreover, this retrospective cohort study was based on the observation and analysis of child and adolescent visual development data obtained from certain areas of Shanghai from 2015 to 2020. The results of school vision screenings and hospital reviews of the study subjects represented the past situation in Shanghai. However, during the follow-up period from 2015 to 2020, there might have been unknown influences (such as the COVID-19 pandemic) affecting the study results, which remained uncertain.

## Conclusion

The timely utilization of ophthalmic clinical services following school vision screenings demonstrated a significantly reduced incidence rate of both myopia and spherical equivalent (SE) progression among preschool children with vision disorders. While the prevalence of vision disorders displays some variation, this study underscores the importance of uplifting the degree of ophthalmic clinical health services utilized by preschool children.

## Data Availability

The datasets generated and analyzed during the current study are available. The materials used in this study are available. Requests for access to the data and materials should be directed to the corresponding author (Huijing Shi, hjshi@fudan.edu.cn).
